# Traditional Chinese medicine classic herbal formula Xiaoqinglong decoction for acute exacerbation of chronic obstructive pulmonary disease: A systematic review protocol

**DOI:** 10.1097/MD.0000000000013761

**Published:** 2018-12-28

**Authors:** Gao Zhen, Jing Jing, Li Fengsen

**Affiliations:** Traditional Chinese Medicine Hospital Affiliated to Xinjiang Medical University & National Clinical Research Base of Traditional Chinese Medicine, Urumqi, China.

**Keywords:** chronic obstructive pulmonary disease, classic herbal formula, clinical efficacy, protocol, systematic review, traditional Chinese medicine, Xiaoqinglong decoction

## Abstract

**Background:**

Xiaoqinglong decoction (XQLD) is 1 of the traditional Chinese medicine (TCM) classic herbal formula that is widely used in Asian for acute exacerbation of chronic obstructive pulmonary disease (AECOPD). In recent years, there has been increasing interest in the use of XQLD to treat COPD in China. So it is necessary to update the research and re-evaluate the efficacy and safety of XQLD to provide up-to-date evidence for COPD management. Therefore, we provide a protocol for a systematic review of XQLD for COPD. This protocol is described for a systematic review to investigate the beneficial effects and safety of XQLD for AECOPD.

**Methods:**

A systematic literature search for article up to October 2018 will be performed in 3 Chinese electronic databases and 2 English electronic databases: Pubmed, Cochrane library, China national knowledge infrastructure (CNKI), Chinese science and technology periodical database (VIP), and Wanfang database. Inclusion criteria are randomized control trials of XQLD in treating AECOPD. The primary outcomes were total clinical efficacy rate, TCM symptom scores, TCM Symptom relief time. The secondary outcome was lung function, blood gas analysis, inflammatory cytokines and C-reactive protein (CRP). The summary results will be pooled using the random-effects model or fixed-effects model according to the heterogeneity of the included studies.

**Result:**

This systematic review will provide an evidence of XQLD for AECOPD, and will submit to a peer-reviewed journal for publication.

**Conclusion:**

The conclusion of this systematic review will provide evidence to judge whether XQLD is an effective intervention for AECOPD patients.

## 1 Introduction

Chronic obstructive pulmonary disease (COPD) is a disorder characterized by progressive airflow limitation caused by chronic inflammation in airways and lung parenchyma. COPD is generally associated with symptoms such as cough, sputum production, and dyspnea.^[1]^ COPD has been a major public health problem in the 21st century,^[2]^ which imposes a substantial economic burden on both patients and government in China. Patients who suffer from COPD may experience cough, dyspnea, chest tightness, and wheezing.^[3,4]^ Chronic cough, sputum production and decreased forced expiratory volume in 1 second (FEV1) have proved to be independently associated with an increased risk of frequent exacerbations and hospitalizations.^[5]^ Currently, treatment of COPD is suboptimal. Many clinical guidelines recommended pharmacological therapies for COPD, but acute exacerbation still occurs frequently and is significantly associated with morbidity and mortality.^[6]^ Thus, many COPD patients resort to complementary and alternative medicine. And Some complementary and alternative therapies may be available and beneficial for COPD.^[7]^

In China and some other Asian counties, traditional Chinese medicine (TCM) has been used more than thousands of years for the treatment of respiratory disease. TCM is a prevalent treatment for COPD, is widely prescribed as an adjunct to western medicine to manage stable COPD in clinical guideline. Although TCM is not the mainstream for treating COPD, it has become increasingly accepted as a form of complementary or complementary medicine in western countries.^[8]^ Studies showed that TCM combines with routine pharmacotherapy are promising benefits on clinical symptoms and quality of life when compared with routine pharmacotherapy alone.^[9]^

Xiaoqinglong decoction (XQLD) was from Zhang Ji's (150–219 CE) Shanghanlun (Treatise on Cold Damage Disorders, just Treatise hereafter), a famous formulary in TCM.^[10]^ And it includes manchurian wildginger (Xixin, Asari Radix et Rhizoma), Pinellia Ternata (Banxia, Pinelliae Rhizoma), liquorice root (Gancao, Glycyrrhizae Radix et Rhizoma),Chinese Magnolcavine Fruit (Wuweizi, Schisandrae Chinensis Fructus), Dried Ginger (Ganjiang, Zingiberis Rhizoma), Cassia Twig (Guizhi, Ramulus Cinnamomi), Chinese Ephedrs Herb *(*mahuang, Ephedrae Herba), White Paeony Root (Baishao, Paeoniae Radix Alba). XQLD has been used against acute airway diseases for thousands of year in ancient China. XQLD was the most commonly used herbal preparation for chronic bronchitis in famous veteran TCM doctor.^[11]^ The therapeutic effect of XQLD in COPD patients has received most traditional Chinese physicians’ approval.^[12]^ So it is necessary for us to assess the efficacy and safety of XQLD, which act as an adjuvant treatment with conventional treatment. The aim of this study is to assess the available evidence of XQLD for COPD according to randomized controlled trials (RCTs).

## 2 Methods

### 2.1 Registration

The study protocol has been registered on international prospective register of systematic review (PROSPERO). The study registration number of PROSPERO is CRD 42018115684. The procedure of this protocol will be conducted according to the preferred reporting items for systematic review and meta-analysis protocols (PROSMA-P) guidance.^[13]^

### 2.2 Inclusion and exclusion criteria

All included trials met the following selection criteria:

1. the study was a randomized controlled trial (RCT);
2. the study examined patients with diagnosed COPD, who received XQLD combined with conventional therapy as treatment compared with those receiving conventional therapy alone;
3. the study included participants irrespective of gender, age, or ethnicity, who was diagnosed with COPD using clearly defined or internationally recognized criteria; and
4. at acute exacerbation stage.

The exclusion criteria were as follows: non-RCTs and quasi-RCTs. Studies were excluded if they did not meet the above eligibility criteria. Additionally, trials with any one of the following conditions were excluded:

1. duplicated publications;
2. case series, reviews, observation study, animal researches, and pharmacological experiments;
3. TCM that were used in both treatment group and control group.
4. Combined with other TCM therapy.

### 2.3 Interventions type

Herbal formulae that must include XQLD was used in the experiment group. There was no limitation on the form of the drug (e.g., liquid, direction, pill, and capsule), dosage, frequency or duration of the treatment. The intervention of control groups included conventional treatment (CT).

### 2.4 Outcome measures

The primary outcomes were total clinical efficacy rate, TCM symptom scores including TCM Syndrome Score (TCMss). Cough symptom score, sputum symptom score, wheezing symptom score, TCM Symptom relief time including Cough relief time, Sputum relief time, wheezing relief time, Lassitude relief time. The secondary outcome was lung function including FEV1, FEV1%pre, and FEV1/ forced vital capacity (FVC). Blood gas analysis including PaO_2_ and PaCO_2_. Inflammatory cytokines including IL-4, IL-8, TNF-α, INF-γ, and CRP.

### 2.5 Search strategy

Pubmed, Cochrane library, China national knowledge infrastructure (CNKI), Chinese science and technology periodical database (VIP), and Wanfang database were retrieved in English or in Chinese by using the following search terms: “(Chronic obstructive pulmonary disease) and (Xiaoqinglong decoction)” or “(Chronic obstructive pulmonary disease) and (Shoseiryuto)”. (Shoseiryuto is a Japanese kampo medicine name of Xiaoqinglong decoction) the search time ranged from the inception of each database until October, 2018. Moreover, we will also manually search the additional relevant studies, using the references of the systematic reviews that published previously.

### 2.6 Selection of studies

Two researchers will scan the titles and summary of the articles they get based on an inclusion criterion that is made previously to eliminate some uncorrelated documents. Besides, for the documents that fit the inclusion criteria, the valuators will read the whole article to make sure if they meet a criterion and prepare to extract relevant information, check the result of the documents brought in. If it meets any diverges, the problem will be solved by consulting another researcher. The lacking information will be replenished by contacting with the writer of the original article.

### 2.7 Data extraction

Two reviewers independently extracted data using a pre-designed collection form. The following data were extracted: general trial characteristics (title, authors, and year); baseline patient and disease data (sample size, age, and gender); interventions (dose, details of control interventions); and outcomes (outcome measures, adverse events). Discrepancies were settled by consensus or a third party. The collected outcome data was inputted into Review Manager 5.3 (RevMan5.3).

### 2.8 Assessment of risk of bias

Criteria for judging the risk of bias were taken from the “risk of bias” assessment tool in The Cochrane Handbook for Systematic Reviews of Interventions 5.1.0.^[14]^ This judgment was evaluated by 2 reviewers independently, and the disagreements were resolved by consulting a third reviewer.

### 2.9 Statistical analysis

Data were analyzed using Review Manager 5.3 software (Cochrane Collaboration, Oxford, UK). Given the characteristics of the extracted data in the review, continuous outcomes were expressed as mean difference (MD) with 95% confidence intervals (CIs). *I*^*2*^ statistics were used to assess heterogeneity. A fixed-effects (FE) model was used if no significant heterogeneity was found in the data (*I*^*2*^ <50%), and a random-effects (RE) model was used if significant heterogeneity was found (*I*^*2*^≥50%). Sensitivity analysis was performed to assess the stability of conclusions. Where heterogeneity was detected, accepted methods were used to explore the statistical heterogeneity using clinical parameters such as treatment duration, sample size, publication year, diagnostic criteria, and publication language. Publication bias was analyzed by funnel plot analysis if sufficient studies (n ≥10) were found.

### 2.10 Quality of evidence

We will also assess the quality of evidence for the main outcomes with the Grading of Recommendations Assessment, Development and Evaluation approach. The 5 items will be investigated, including limitations in study design, inconsistency, inaccuracies, indirectness, and publication bias.

### 2.11 Description of possible mechanisms

Animal-based mechanism studies of XQLD and related autoimmune disease will be searched.

### 2.12 Ethics and dissemination

This systematic review will not require ethical approval because there are no data used in our study that are linked to individual patient data. In addition, findings will be disseminated through peer-review publications.

## 3 Discussion

TCM classic herb formulas Xiaoqinglong decoction have been recommended as complementary and alternative regimens for respiratory system including cough, asthma, COPD,^[12]^ and allergic airway disease in Asian counties including China, Japan^[15]^ for a long time. Especially in China. Studies showed that XQLD was an effective drug for COPD treatment and its function was related to gene expression alteration,^[12]^ it can inhibits the progress of COPD via attenuating the autophagy process.^[16]^

This study focuses on evaluating the efficacy and safety of XQLD in combination of CT for treating COPD compared with CT alone. It may help to propose the clinical recommendation for AECOPD patients and to provide more reliable evidence for XQLD's application.

Studies have predicted that paeonol, glycyrrhizin, and geranidin are the 3 most effective ingredients in XQLD for cough and asthma.^[17]^ XQLD could improve the airway hyperreactivity and airway reconstruction, remiss the inflammation of COPD airway.^[18]^ Oral administration of XQLD (Shoseiryuto) reduced the production of IL-4 and IL-5 in airway inflammatory model mice;^[19]^ increase FoxP3 + , CD4 + (BALB/c mice).^[20]^ Rectify imbalance of oxidation/anti-oxidation and alleviate inflammatory reactions,^[10]^ exhibited higher levels of apoptosis, up-regulate LC3II/LC3I ratio and down-regulate p62 level,^[21]^ NF-κB^[22]^, and γ-GCS^[23]^ level in COPD rats. One experimental research^[24]^ showed that there are no side effects such as interstitial pneumonia, myopathy, or impaired liver function.

## Author contributions

**Conceptualization:** Zhen Gao.

**Data curation:** Zhen Gao.

**Formal analysis:** Zhen Gao, Jing Jing.

**Funding acquisition:** Zhen Gao.

**Project administration:** Li Fengsen.

**Resources:** Zhen Gao.

**Software:** Zhen Gao.

**Supervision:** Zhen Gao.

**Visualization:** Zhen Gao, Jing Jing.

**Writing - original draft:** Zhen Gao.

**Writing - review & editing:** Zhen Gao, Li Fengsen.
